# Supplementary material for: Multi‐dataset identification of innovative feature genes and molecular mechanisms in keratoconus
Source: J Cell Mol Med. 2024 Sep 19;28(18):e70079. doi: 10.1111/jcmm.70079 (PMC11412914; doi:10.1111/jcmm.70079)
Supplement: Supplementary file 2 — Material S2. [file JCMM-28-e70079-s002.zip › 7.GSEA analysis/6.SNORA74B/KEGG_CELL_CYCLE.html]

Details for gene set KEGG\_CELL\_CYCLE[GSEA]

|  || Dataset | SNORA74B.group.cls#h\_versus\_l |
| Phenotype | group.cls#h\_versus\_l |
| Upregulated in class | l |
| GeneSet | KEGG\_CELL\_CYCLE |
| Enrichment Score (ES) | -0.4241986 |
| Normalized Enrichment Score (NES) | -1.5188625 |
| Nominal p-value | 0.09780439 |
| FDR q-value | 0.322674 |
| FWER p-Value | 0.742 |
Table: GSEA Results Summary

  

Fig 1: Enrichment plot: KEGG\_CELL\_CYCLE      
 Profile of the Running ES Score & Positions of GeneSet Members on the Rank Ordered List

  

| SYMBOL | TITLE | RANK IN GENE LIST | RANK METRIC SCORE | RUNNING ES | CORE ENRICHMENT || 1 | CDC14A | na | 1982 | 0.372 | -0.0320 | No |
| 2 | ANAPC1 | na | 6932 | 0.164 | -0.1638 | No |
| 3 | CCNE2 | na | 7159 | 0.158 | -0.1596 | No |
| 4 | ESPL1 | na | 7434 | 0.150 | -0.1573 | No |
| 5 | E2F2 | na | 7913 | 0.138 | -0.1616 | No |
| 6 | PRKDC | na | 7979 | 0.137 | -0.1542 | No |
| 7 | CCNA1 | na | 8695 | 0.120 | -0.1667 | No |
| 8 | CCNE1 | na | 8992 | 0.114 | -0.1675 | No |
| 9 | STAG2 | na | 9734 | 0.098 | -0.1822 | No |
| 10 | ATM | na | 9797 | 0.097 | -0.1774 | No |
| 11 | CDKN2B | na | 10073 | 0.091 | -0.1792 | No |
| 12 | CREBBP | na | 10525 | 0.083 | -0.1866 | No |
| 13 | ATR | na | 10571 | 0.082 | -0.1823 | No |
| 14 | YWHAE | na | 11170 | 0.072 | -0.1946 | No |
| 15 | SMC1A | na | 11202 | 0.071 | -0.1907 | No |
| 16 | MDM2 | na | 11211 | 0.071 | -0.1860 | No |
| 17 | CDK1 | na | 11443 | 0.068 | -0.1881 | No |
| 18 | ORC5 | na | 11738 | 0.064 | -0.1923 | No |
| 19 | TGFB2 | na | 11803 | 0.063 | -0.1898 | No |
| 20 | FZR1 | na | 12438 | 0.054 | -0.2045 | No |
| 21 | STAG1 | na | 12632 | 0.051 | -0.2065 | No |
| 22 | SMAD4 | na | 13214 | 0.045 | -0.2203 | No |
| 23 | SMAD2 | na | 13222 | 0.045 | -0.2175 | No |
| 24 | SMC1B | na | 13265 | 0.044 | -0.2157 | No |
| 25 | RB1 | na | 13606 | 0.040 | -0.2227 | No |
| 26 | BUB3 | na | 13898 | 0.037 | -0.2286 | No |
| 27 | RBX1 | na | 14044 | 0.035 | -0.2304 | No |
| 28 | YWHAZ | na | 14437 | 0.031 | -0.2396 | No |
| 29 | ANAPC5 | na | 15115 | 0.023 | -0.2576 | No |
| 30 | CDC23 | na | 15160 | 0.023 | -0.2573 | No |
| 31 | ANAPC7 | na | 15214 | 0.022 | -0.2573 | No |
| 32 | CCND1 | na | 15223 | 0.022 | -0.2560 | No |
| 33 | YWHAB | na | 15465 | 0.020 | -0.2616 | No |
| 34 | PTTG2 | na | 15583 | 0.019 | -0.2638 | No |
| 35 | ANAPC4 | na | 15647 | 0.018 | -0.2643 | No |
| 36 | SMAD3 | na | 16318 | 0.011 | -0.2829 | No |
| 37 | PTTG1 | na | 16518 | 0.009 | -0.2881 | No |
| 38 | HDAC1 | na | 16993 | 0.005 | -0.3014 | No |
| 39 | E2F3 | na | 17092 | 0.004 | -0.3040 | No |
| 40 | ORC2 | na | 17284 | 0.002 | -0.3094 | No |
| 41 | ANAPC13 | na | 17316 | 0.001 | -0.3102 | No |
| 42 | TFDP1 | na | 17332 | 0.001 | -0.3106 | No |
| 43 | ANAPC11 | na | 17447 | 0.000 | -0.3138 | No |
| 44 | GSK3B | na | 17871 | -0.004 | -0.3258 | No |
| 45 | CDC16 | na | 17914 | -0.005 | -0.3266 | No |
| 46 | SKP2 | na | 17943 | -0.005 | -0.3271 | No |
| 47 | SKP1 | na | 18228 | -0.009 | -0.3347 | No |
| 48 | ORC4 | na | 18318 | -0.010 | -0.3366 | No |
| 49 | CDKN2D | na | 18557 | -0.012 | -0.3426 | No |
| 50 | RBL2 | na | 18808 | -0.015 | -0.3489 | No |
| 51 | PCNA | na | 19297 | -0.021 | -0.3615 | No |
| 52 | SFN | na | 19485 | -0.023 | -0.3654 | No |
| 53 | TP53 | na | 19572 | -0.024 | -0.3663 | No |
| 54 | CDKN2A | na | 19773 | -0.026 | -0.3703 | No |
| 55 | GADD45G | na | 19816 | -0.026 | -0.3697 | No |
| 56 | CDKN1B | na | 20194 | -0.030 | -0.3785 | No |
| 57 | YWHAG | na | 20368 | -0.032 | -0.3813 | No |
| 58 | CUL1 | na | 20440 | -0.033 | -0.3811 | No |
| 59 | CCND2 | na | 21016 | -0.040 | -0.3949 | No |
| 60 | YWHAQ | na | 21272 | -0.043 | -0.3994 | No |
| 61 | CCNH | na | 21370 | -0.044 | -0.3992 | No |
| 62 | CDC20 | na | 21595 | -0.047 | -0.4024 | No |
| 63 | MAD2L1 | na | 21747 | -0.049 | -0.4035 | No |
| 64 | RBL1 | na | 21873 | -0.050 | -0.4037 | No |
| 65 | CDC27 | na | 21919 | -0.051 | -0.4015 | No |
| 66 | CDC14B | na | 21922 | -0.051 | -0.3981 | No |
| 67 | HDAC2 | na | 22146 | -0.053 | -0.4009 | No |
| 68 | DBF4 | na | 22665 | -0.060 | -0.4118 | No |
| 69 | ORC3 | na | 22775 | -0.062 | -0.4107 | No |
| 70 | CDK7 | na | 22835 | -0.063 | -0.4081 | No |
| 71 | RAD21 | na | 23132 | -0.067 | -0.4121 | No |
| 72 | ANAPC10 | na | 23493 | -0.073 | -0.4176 | No |
| 73 | MCM3 | na | 23664 | -0.076 | -0.4173 | No |
| 74 | ABL1 | na | 23665 | -0.076 | -0.4122 | No |
| 75 | CCNB3 | na | 24048 | -0.082 | -0.4177 | No |
| 76 | ANAPC2 | na | 24147 | -0.083 | -0.4148 | No |
| 77 | CDK4 | na | 24176 | -0.084 | -0.4100 | No |
| 78 | CCNB2 | na | 24670 | -0.092 | -0.4179 | Yes |
| 79 | CCNB1 | na | 24727 | -0.093 | -0.4131 | Yes |
| 80 | PKMYT1 | na | 24796 | -0.095 | -0.4087 | Yes |
| 81 | CDC26 | na | 24971 | -0.098 | -0.4070 | Yes |
| 82 | TFDP2 | na | 25023 | -0.099 | -0.4017 | Yes |
| 83 | MCM7 | na | 25197 | -0.102 | -0.3998 | Yes |
| 84 | CCND3 | na | 25274 | -0.103 | -0.3950 | Yes |
| 85 | EP300 | na | 25385 | -0.105 | -0.3910 | Yes |
| 86 | MYC | na | 25534 | -0.108 | -0.3879 | Yes |
| 87 | MAD2L2 | na | 25644 | -0.110 | -0.3836 | Yes |
| 88 | ZBTB17 | na | 26303 | -0.123 | -0.3942 | Yes |
| 89 | SMC3 | na | 26515 | -0.128 | -0.3916 | Yes |
| 90 | MAD1L1 | na | 26656 | -0.131 | -0.3867 | Yes |
| 91 | GADD45A | na | 26779 | -0.134 | -0.3810 | Yes |
| 92 | CHEK2 | na | 26873 | -0.137 | -0.3744 | Yes |
| 93 | MCM4 | na | 27082 | -0.141 | -0.3708 | Yes |
| 94 | CDK2 | na | 27098 | -0.141 | -0.3616 | Yes |
| 95 | E2F1 | na | 27392 | -0.149 | -0.3599 | Yes |
| 96 | CDC25B | na | 27564 | -0.153 | -0.3544 | Yes |
| 97 | CDK6 | na | 27632 | -0.155 | -0.3458 | Yes |
| 98 | CDKN2C | na | 27731 | -0.158 | -0.3379 | Yes |
| 99 | WEE1 | na | 28113 | -0.168 | -0.3375 | Yes |
| 100 | GADD45B | na | 28844 | -0.190 | -0.3456 | Yes |
| 101 | CDC7 | na | 28938 | -0.193 | -0.3352 | Yes |
| 102 | BUB1 | na | 29117 | -0.198 | -0.3268 | Yes |
| 103 | CCNA2 | na | 29161 | -0.199 | -0.3145 | Yes |
| 104 | E2F4 | na | 29168 | -0.200 | -0.3010 | Yes |
| 105 | TTK | na | 29420 | -0.207 | -0.2942 | Yes |
| 106 | MCM5 | na | 29445 | -0.208 | -0.2807 | Yes |
| 107 | CDKN1C | na | 29574 | -0.212 | -0.2700 | Yes |
| 108 | TGFB1 | na | 30144 | -0.231 | -0.2707 | Yes |
| 109 | MCM6 | na | 30422 | -0.242 | -0.2622 | Yes |
| 110 | YWHAH | na | 30773 | -0.257 | -0.2548 | Yes |
| 111 | E2F5 | na | 30880 | -0.262 | -0.2400 | Yes |
| 112 | MCM2 | na | 31109 | -0.274 | -0.2280 | Yes |
| 113 | CDKN1A | na | 31225 | -0.280 | -0.2122 | Yes |
| 114 | TGFB3 | na | 31437 | -0.291 | -0.1985 | Yes |
| 115 | CDC25A | na | 31451 | -0.292 | -0.1790 | Yes |
| 116 | CDC45 | na | 31672 | -0.305 | -0.1646 | Yes |
| 117 | CHEK1 | na | 32354 | -0.344 | -0.1608 | Yes |
| 118 | CDC25C | na | 33044 | -0.398 | -0.1536 | Yes |
| 119 | BUB1B | na | 33403 | -0.439 | -0.1340 | Yes |
| 120 | PLK1 | na | 33434 | -0.441 | -0.1048 | Yes |
| 121 | ORC6 | na | 33481 | -0.447 | -0.0757 | Yes |
| 122 | WEE2 | na | 33676 | -0.474 | -0.0490 | Yes |
| 123 | CDC6 | na | 33771 | -0.489 | -0.0184 | Yes |
| 124 | ORC1 | na | 34455 | -0.675 | 0.0079 | Yes |
Table: GSEA details [plain text format]

  

Fig 2: KEGG\_CELL\_CYCLE      
 Blue-Pink O' Gram in the Space of the Analyzed GeneSet

  

Fig 3: KEGG\_CELL\_CYCLE: Random ES distribution      
 Gene set null distribution of ES for **KEGG\_CELL\_CYCLE**
